# Temperature affects major fatty acid biosynthesis in noug (*Guizotia abyssinica*) self-compatible lines

**DOI:** 10.3389/fnut.2024.1511098

**Published:** 2024-12-13

**Authors:** Adane Gebeyehu, Cecilia Hammenhag, Kassahun Tesfaye, Rodomiro Ortiz, Mulatu Geleta

**Affiliations:** ^1^Department of Plant Breeding, Swedish University of Agricultural Sciences, Alnarp, Sweden; ^2^Bio and Emerging Technology Institute, Addis Ababa, Ethiopia; ^3^Institute of Biotechnology, Addis Ababa University, Addis Ababa, Ethiopia

**Keywords:** fatty acid composition, *Guizotia abyssinica*, noug, oleic acid, self-compatibility, temperature

## Abstract

**Introduction:**

Noug (*Guizotia abyssinica*) is an economically important edible oilseed crop in Ethiopia with a large variation in seed set, seed oil content, and fatty acid composition among populations. Although noug is generally strictly self-incompatible (SI), self-compatible (SC) lines were recently developed. This study was conducted to investigate the levels of variation in seed setting and oleic acid content among the self-compatible lines.

**Methods:**

The starting materials for the seed setting study were 200 genotypes selected from 100 inbred lines and having, on average, 57 seeds per capitulum, which is higher than that of the SI populations. The SC genotypes were analyzed for their oleic acid content using the half-seed technique.

**Results:**

The analysis of 20 SC lines revealed a high variation in oleic acid content with 70% of the SC lines having 20% or more oleic acid after they were grown under 25°C/21°C day/night temperatures (high-temperature treatment). The oleic acid content increased from 8.2% before to 22.5%, on average, after the high-temperature experiment in the greenhouse. In contrast, the percentage of oleic acid in these lines grown at 21°C/18°C day/night temperatures decreased from 8.2% to 4.4% on average. There was a highly significant positive correlation between oleic acid content and temperature in SC lines.

**Conclusion:**

The study suggests a significant contribution of genotype to the variation in seed setting and environmental factors (mostly temperature) to the oleic acid content. The noug SC-lines showed highly significant variation in seed setting and oleic acid content, which could be used for improving the crop’s seed yield and oil quality.

## 1 Introduction

Edible oils are essential bioactive compounds found in the majority of food types prepared from plant and animal products. Oilseed crops are sources of edible oils that have concentrated calories, essential vitamins, proteins, and micronutrients (1–3). Hence, oilseed crops are widely grown and used in the developing world by small-scale farmers. Currently, a few crop species, such as oilseed rape (*Brassica napus*), sunflower (*Helianthus annuus*), and soybean (*Glycine max*), dominate the global edible oil seed market. However, the demand for high-quality seed oil continues to increase due to the ever-growing population. To meet the ever-growing demand for food oils, it is necessary to diversify and improve the sources by increasing the production of major oil crops, as well as minor oilseed crops, such as noug (*Guizotia abyssinica*) through breeding.

Noug is the second most important edible oilseed crop in Ethiopia, which is produced primarily for its edible oil, although its seed is also rich in protein (3–5). In Ethiopia, half of the produce is crushed for oil extraction and the rest is used for confectionery and food purposes (6). Noug seed oil supplies about 60% of the edible oil required in Ethiopia and just 3% of the edible oil requirement in India (7–9). It is a major oilseed crop that plays an important role in improving the rural livelihoods of millions of Ethiopians (5, 10). Noug is cultivated in a total area of 358,828 ha (31.1% of the total area dedicated to oilseeds) with a total production of 295,000 MT in Ethiopia (11–13). Although Ethiopia and India are the major noug producers, it is cultivated in several other African and Asian countries on a small-scale basis (8, 14).

Noug is generally a strictly out-crossing species with sporophytic self-incompatibility (10, 15), which is a type of homomorphic self-incompatibility. It requires insect pollinators for cross-pollination, being bees as its main pollinators (10). Self-incompatibility (SI) is disadvantageous in noug breeding, as it complicates breeding efforts to develop cultivars of interest through either free intercrosses or selfing (8, 16). In areas where the pollinators are not sufficiently available, the seed set is low in noug resulting in lower seed yield, with SI being a major contributing factor (4, 17). In this regard, self-compatibility (SC) has various advantages over SI in this crop, as it allows self-pollination that facilitates a full seed set and hence potentially higher seed yield without the need for insect pollinators. In addition, SC facilitates the development of recombinant inbred lines (RILs) and pure lines with desirable characteristics of various traits, such as high seed and oil yields, high oleic acid oil, and increased resistance to pathogens and pests. Self-compatible lines were developed from the Ethiopian noug gene pool to utilize these advantages for improving the crop (5, 15).

The noug oil content was previously reported to range between 29 and 39% (7), 30 and 35% (18), 42 and 44% (19), and 27 and 56% (1). The most abundant fatty acid in noug is linoleic acid, which makes up more than 60% of all fatty acids (7, 19, 20). The Ethiopian noug seed oil contains 51 to 80% linoleic acid (1–3), while the remaining percentage accounts for oleic, palmitic, stearic, and other unsaturated fatty acids. The oleic acid content in the seed oil was also reported to be less than 13% of the total fatty acids in the Ethiopian noug (19, 21, 22) while it ranges from 5 to 25% of the total fatty acids in Indian noug (23). However, a few Ethiopian noug landrace populations with oleic acid content above 13% have been found (1, 2). Crossbreeding of selected genotypes from these landrace populations significantly increased the oleic acid content with some breeding lines having an oleic acid content of above 80% under greenhouse conditions (1, 2). However, oil content and fatty acid composition may vary depending on the maturity level of the seeds and the origin of the materials (9, 24).

Due to the demand for high oleic acid edible oil, plant breeders have developed many oilseed cultivars with high oleic acid content. In line with this, there is a growing demand for noug oil with an increased oleic-to-linoleic acid ratio. Hence, developing noug cultivars with a high oleic acid content has emerged as a key breeding objective. However, research has shown that temperature affects the oleic acid levels in seed oil in different oilseed crops, including sunflower (25), flax (26), soybean (27), oilseed rape (28) and safflower (29). Research on noug also showed strong environmental effects on the oleic acid content of its oil (1, 3). Research investigating the effect of temperature on the fatty acid composition of noug seed oil could provide insight into the extent to which oleic acid is affected by temperature. The present study aimed at analyzing the fatty acid composition, determining the effects of temperature on oleic acid levels in the seed oil, and evaluating the variation in seed setting of noug SC-lines. The study also aimed to identify SC-lines with high oleic acid content and are efficient at setting self-seeds that could be utilized for developing high-yielding inbred cultivars rich in oleic acid.

## 2 Materials and methods

### 2.1 Plant material

Most of the genotypes were selected from breeding populations bred for desirable traits such as self-compatibility, early maturity, less sensitivity to photoperiod, and high oil content. For the seed-setting experiment, 94 SC genotypes representing different breeding lines were used. Based on their pedigree, these genotypes were grouped into 27 clusters, each comprising one to nine genotypes. Seventeen of the 94 genotypes were selected from SC-lines that were tested only under greenhouse conditions where photosynthetic photon flux density (PPFD), photoperiod, temperature, and relative humidity were monitored and kept at 90 ± 3 μmol m^−2^ s^−1^, 16 h d^−1^, 20 ± 0.5°C, and 65% ± 5%, respectively. The remaining 77 genotypes were selected from breeding lines that were tested under field conditions at different locations in Ethiopia. The final field trial was conducted from July to December 2018 at Holeta Agricultural Research Center, located 30 km west of Addis Ababa at 2400 masl, 9^o^00′ N, and 38^o^30′ E having a soil type of nitosols and vertisols. Hence, the 77 genotypes were sourced from seeds harvested from this field trial. The experimental site receives an average of 1,144 mm annual rainfall and experiences average temperatures ranging from 6°C to 22°C. For the fatty acid composition analysis, 75 genotypes comprising both self-compatible and self-incompatible lines, were used. These genotypes (except one) were selected from harvests of a concurrent field trial at the same location.

### 2.2 Greenhouse experiments

For all experiments, seeds of each line were planted at two replications and grown in 2.5 L plastic pots filled with soils in a greenhouse at the Swedish University of Agricultural Sciences (SLU), Alnarp, Sweden.

#### 2.2.1 The self-seed set experiment

For the self-seed set experiment, plants were grown in the greenhouse from December 2019 to May 2020 in 16 h of daylight where the day/night temperatures were set to 25°C/21°C, respectively. In this experiment, 11 phenological and agromorphological traits were measured. These traits are days to flowering (DTF), plant height (PH), flower size (FS), capitulum size (CS), ray floret color (RFC), pollen color (PC), number of seeds per capitulum (NSPC), thousand seed weight (TSW), seed color (SC), seed size (SS), and seed shape (SSH). The descriptions of these traits are provided in Supplementary Table S1. The data on seed traits (NSPC, TSW, SC, SS, and SSH) were collected following the harvesting of mature seeds in May 2020 and subsequent cleaning. Clean seeds from 10 representative capitula of each genotype were counted using the Contador seed counter (Pfeuffer GmbH, Germany^1^;) to determine the NSPC for each SC genotype. This was followed by weighing the seeds using a precision balance to determine the TSW of the genotypes.

#### 2.2.2 Experiments on the effects of temperature on the seed oil fatty acid profiles

Two experiments were carried out on the same 75 genotypes to determine the effects of temperature on their seed oil fatty acid profiles. The two experiments differed in the day/night temperatures in a greenhouse chamber where the plants were grown until harvest. The experiment conducted under 25°C/21°C day/night temperatures is referred to as the “high-temperature experiment” whereas the experiment conducted under 21°C/18°C day/night temperatures is referred to as the “low-temperature experiment.” For these experiments, the half-seed method was applied where the seeds of the target genotypes were cross-sectionally cut into halves with a sharp scalpel. The embryo-containing halves were then planted in soil-filled pots and grown in a greenhouse while the other halves were weighed separately using a precision balance, followed by oil extraction, and the fatty acid profile was analyzed using gas chromatography (GC).

For the high-temperature experiment, plants that emerged from the half-seeds were grown in the greenhouse from February to June 2020. The oil analysis of the parental half-seeds was carried out from January to February 2020. Then, mature seeds were harvested in June 2020 and the oil analysis of the progeny seeds was carried out from October to January 2020. For the low-temperature experiment, plants that emerged from the half-seeds were grown in the greenhouse from September 2020 to February (13). The oil analysis of the parental half-seeds was carried out in September 2020. Then, mature progeny seeds were harvested in February (13) and their oil analysis was carried out in May (13). For the high-temperature experiment, data were collected from all 75 genotypes. Unfortunately, only 20 of the 75 genotypes used for the low-temperature experiment provided mature healthy seeds while the others failed due to a sudden disease attack. Hence, the two experiments were compared only based on the 20 common genotypes.

### 2.3 Oil extraction and methylation of fatty acids

Total lipids from noug seeds were extracted according to Bligh and Dyer (30). For major fatty acid analysis of the progeny seeds, 10 seeds were homogenized in a mixture of 3.75 mL of methanol: chloroform (2:1, v/v) and 1 mL 0.15 M acetic acid using the Ultra Turrax seed homogenizer for approximately 2 min. This was followed by adding 1.25 mL chloroform and 0.9 mL Millipore water and thoroughly mixing by vortexing. Each of the aforementioned reagents was reduced by half for the half-seed analysis of parental seeds. The mixture was centrifuged for 2 min at 3000 rpm for phase separation. Then, 1 mL of the bottom phase was transferred to new screw cap glass tubes and evaporated under a bath of sand with a weak beam of nitrogen gas heated to 70°C. Thereafter, 2 mL H_2_SO_4_ (2% in absolute methanol) was added and the fatty acids were methylated at 90°C for 45 min. After the solution was cooled down to room temperature, 200 μL of 200 nmol methyl-heptadecanoate (17:0-ME; artificial fatty acid) was added to the fatty acid methyl esters (FAMEs) as an internal standard. To this, 2 mL hexane and 1 mL Millipore water were added and thoroughly mixed before centrifugation for 2 min at 2000 rpm. The last step was transferring 200 μL of the hexane phase to the GC vials for oleic acid and other major fatty acids analysis.

### 2.4 Fatty acids profiling by gas chromatography

Separation of the FAMEs was done using an Agilent (model 7890A) gas chromatograph (Agilent Technologies, Inc., United States) equipped with an automatic Agilent Pal Injector (API) and automatic liquid sampler (ALS) having 7693A interface on 7890A, a WCOT Fused Silica CP-Wax 58 column and a flame ionization detector (FID). Four microliters of the heptane extract of the samples and the external standard were injected into the stainless-steel capillary column (105 m × 0.530 mm). The column temperature was held at 150°C for 30 s, increased at a rate of 6°C/min to 250°C and held there for 2.4 min. The carrier and the detector gasses were maintained at 240°C and 270°C, respectively. The FAMEs were identified by comparing the retention time with the standards and peak integration was performed using the GC calculators using the 17:0-ME peak area as a reference. All detectable fatty acids were identified although palmitic acid (C16:0), stearic acid (C18:0), oleic acid (C18:1), and linoleic acid (C18:2) were targeted in this study.

### 2.5 Statistical analysis

The proportion of each fatty acid in the oil was computed as the ratio of their weight to the total fatty acids based on the GC peak area for the different FAMEs using the GC Calculators Instrument 1 software. In this study, palmitic, stearic, oleic, and linoleic acids were analyzed as they are the major fatty acids in noug seed oil. Due to the insignificant variation between the samples, minor fatty acids were excluded from the analysis. The following procedure was followed to calculate the area fraction X*i* of the individual fatty acid methyl esters expressed as a percentage per area of methyl esters as:


$$Xi=\frac{\mathrm{Ai}}{\sum A}x100$$

where A*i* is the area of individual fatty acid methyl esters and ∑A is the sum of areas under the peaks of all individual fatty acid methyl esters. Boxplot analysis, analysis of variance (ANOVA), Pearson’s correlation analysis, paired *t*-test, and Tukey pairwise comparison were conducted using MINITAB 18.1.0.0 statistical software.^2^

## 3 Results

### 3.1 Phenotypic trait variation and correlations between quantitative traits

**Figure 1:**
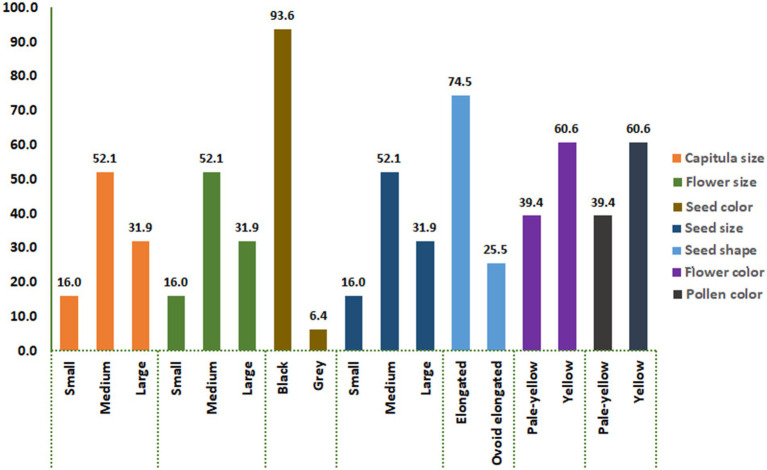
The percentage distribution of SC lines across different classes of seven categorical traits, including capitulum size, flower size, seed color, seed size, seed shape, flower color, and pollen color. Each bar represents the proportion of SC lines within the corresponding trait category.

**Figure 2:**
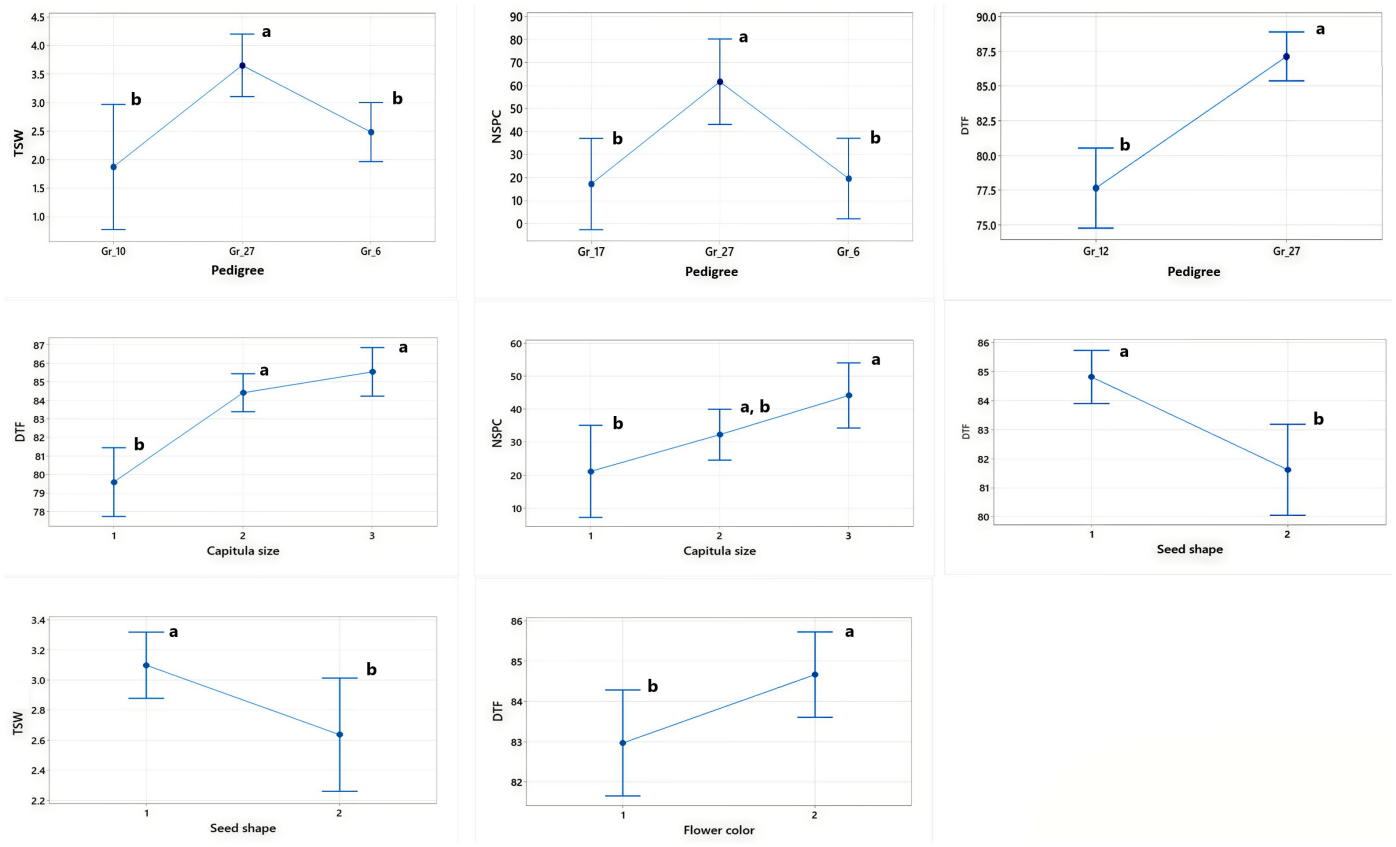
Interval plots showing pairwise comparisons between categorical traits (capitulum size (CS), seed shape (SSH), and flower color (FC) and pedigree versus qualitative traits (thousand seed weight (TSW), number of seeds per capitulum (NSPC), and days to flowering (DTF)) that showed significant differences when tested using the Tukey method. Different letters above the bars within each box indicate significant differences within the same trait group.

The analysis of variance (ANOVA) revealed highly significant differences (*p* ≤ 0.01) among the SC-lines in most quantitative traits (Table 1). A wide variability was observed for DTF, TSW, PH, and NSPC. There was a significant variation in seed setting between the 94 SC-lines (Supplementary Table S2). The number of seeds per capitulum ranged from 1 to 113, with an average seed set of 57 seeds per capitulum. The SC-line CB1-14 had the highest number of seeds (113 seeds/capitulum) followed by CB2-3SFSP (110 seeds/capitulum) and CB8-7 (102 seeds/capitulum) respectively. The lowest number of seeds (1 seed/capitulum) were obtained from CB3-9 and CB4-8 (Supplementary Table S2). Large differences in the percentage distribution of SC-lines across different classes of seven qualitative/categorical traits (CS, FS, SC, SS, SSH, FC, and PC) were observed. For instance, 93.6% of SC-lines produced black seeds while only 6.4% produced grey seeds (Figure 1). Pearson’s correlation coefficients between all pairs of quantitative traits of the SC-lines are shown in Table 2. DTF, NSPC, and TSW exhibited a significant positive correlation with each other, with NSPC showing a particularly strong positive correlation with TSW. Interestingly, PH did not correlate with all quantitative traits tested. A weak but significant positive correlation was observed between TSW and NSPC (Table 2). In the present study, 35% of the SC-lines had 40 or more seeds per capitulum (Supplementary Table S2). In this study, significant differences were found in TSW, NSPC, and DTF between SC-lines of different pedigrees. In addition, significant differences were observed in TSW, NSPC, and DTF between SC-lines belonging to different classes of capitulum size, seed shape, and flower color (Figure 2).

**Table 1:** Analysis of variance for four quantitative traits by grouping the self-compatible genotypes according to their pedigree, capitula size, seed color, seed shape, and flower color.

Trait	SV	DF	Adj SS	Adj MS	*F*-value	*p*-value	SD pairs	Trait	SV	DF	Adj SS	Adj MS	F-Value	P-Value	SD pairs
DTF	Pedigree	13	347.8	26.75	2.07	0.029*	12 vs. 27	NSPC	Seed color	1	949.8	949.8	1.21	0.274	None
	Error	62	800.7	12.92					Error	92	72199.3	784.8			
	Total	75	1148.5						Total	93	73149.1				
PH	Pedigree	13	16,103	1238.7	1.83	0.058	None	TSW	Seed color	1	1.678	1.6779	1.91	0.17	None
	Error	62	41,954	676.7					Error	92	80.837	0.8787			
	Total	75	58,057						Total	93	82.515				
NSPC	Pedigree	13	23,576	1813.5	3.16	0.001**	6 vs. 27	DTF	Seed shape	1	181.8	181.79	12.22	0.001**	1 vs. 2
	Error	62	35,580	573.9			17 vs. 27		Error	92	1368.2	14.87			
	Total	75	59,156						Total	93	1,550				
TSW	Pedigree	13	17.86	1.3739	2.18	0.021*	6 vs. 27	PH	Seed shape	1	11	11	0.01	0.905	None
	Error	62	39.07	0.6302			19 vs. 27		Error	92	70982.7	771.55			
	Total	75	56.93						Total	93	70993.7				
DTF	Capitula size	2	369.1	184.55	14.22	0**	1 vs. 2	NSPC	Seed shape	1	4,392	4,392	5.88	0.017*	1 vs. 2
	Error	91	1180.9	12.98			1 vs. 3		Error	92	68,757	747.4			
	Total	93	1,550						Total	93	73,149				
PH	Capitula size	2	1,489	744.4	0.97	0.381	None	TSW	Seed shape	1	3.79	3.7898	4.43	0.038*	1 vs. 2
	Error	91	69,505	763.8					Error	92	78.725	0.8557			
	Total	93	70,994						Total	93	82.515				
NSPC	Capitula size	2	5,705	2852.6	3.85	0.025*	1 vs. 3	DTF	Flower color	1	64.36	64.36	3.99	0.049*	1 vs. 2
	Error	91	67,444	741.1					Error	92	1485.64	16.15			
	Total	93	73,149						Total	93	1,550				
TSW	Capitula size	2	4.283	2.1415	2.49	0.088	None	PH	Flower color	1	72.5	72.48	0.09	0.76	None
	Error	91	78.232	0.8597					Error	92	70921.2	770.88			
	Total	93	82.515						Total	93	70993.7				
DTF	Seed color	1	2.85	2.848	0.17	0.682	None	NSPC	Flower color	1	13.8	13.8	0.02	0.895	None
	Error	92	1547.15	16.817					Error	92	73135.3	794.95			
	Total	93	1,550						Total	93	73149.1				
PH	Seed color	1	1,264	1263.5	1.67	0.2	None	TSW	Flower color	1	0.1444	0.1444	0.16	0.689	None
	Error	92	69,730	757.9					Error	92	82.3702	0.8953			
	Total	93	70,994						Total	93	82.5146				

DTF = Days to flowering; PH = Plant height; NSPC = Number of seeds per capitulum; TSW = Thousand seed weight; SV = Source of variation; DF = Degrees of freedom; SD pairs = significantly different pairs (*p* < 0.05) as per Tukey Pairwise Comparisons. * = 0.01 < *p* < 0.05; ** = *p* < 0.01. Note: Because flower size and seed size were 100% correlated with capitula size, and pollen color was 100% correlated with flower color, we did not group the quantitative traits by flower color, seed size, and pollen color for the analysis of variance.

**Table 2:** Pearson correlation coefficient between days to flowering (DTF), plant height (PH), number of seeds per capitulum (NSPC), and thousand seed weight (TSW).

		DTF	PH	NSPC
PH	*r*	−0.19		
	*p*-value	0.066		
NSPC	*r*	0.204	−0.025	
	*p*-value	0.048*	0.809	
TSW	*r*	0.233	−0.104	0.825
	*p*-value	0.024*	0.318	< 0.001***

*r* = Pearson’s correlation coefficient * and *** indicate a significant *r* at *p* < 0.05, and *P* < 0.001, respectively.

### 3.2 Major fatty acids composition analysis

**Figure 3:**
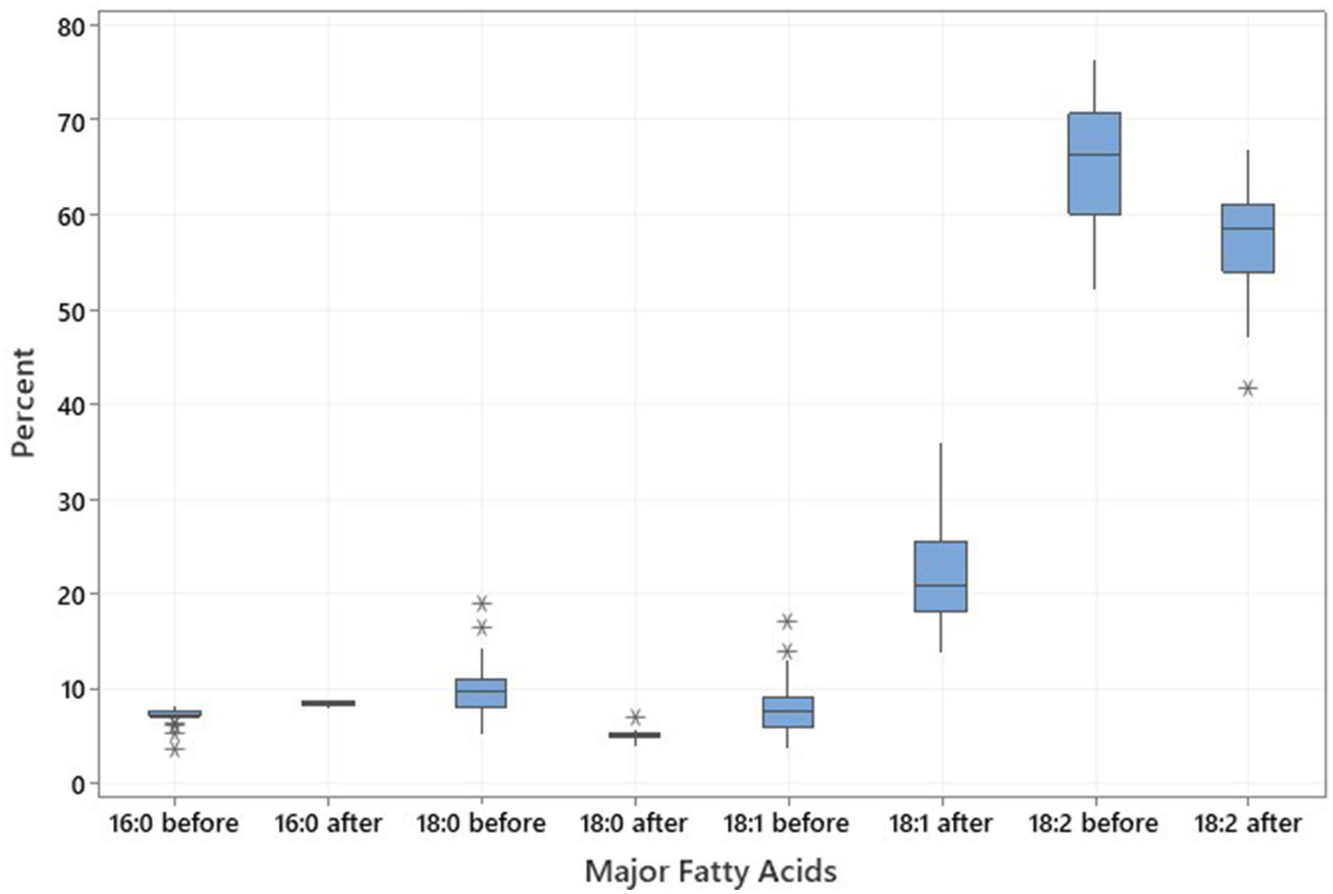
Boxplot illustrating the distribution and variation in major fatty acids palmitic acid (C16:0), stearic acid (C18:0), oleic acid (C18:1), and linoleic acid (C18:2) across the 20 noug self-compatible (SC)-lines, illustrating the changes in fatty acid profiles resulting from exposure to high temperatures.

Twenty noug SC-lines were evaluated for their fatty acid composition focusing on the four major fatty acids; namely, palmitic acid (C16:0), stearic acid (C18:0), oleic acid (C18:1), and linoleic acid (C18:2). The plants were grown in greenhouse conditions within a high-temperature range between 21°C to 25°C. Before the greenhouse experiment, oleic acid content ranged from 3.78% (CB4-10) to 17.03% (NG143D), increasing post-experiment to a range from 13.90% (NG109) to 36.05% (CB4-2). Palmitic acid levels ranged from 3.56% (NG143D) to 8.24% (NG135A) before the greenhouse experiment and increased to range from 7.95% (CB4-13) to 9.00% (NG123) after. In contrast, stearic acid content ranged from 5.29% (CB4-10) to 19.04% (NG142B) before the experiment and decreased to range from 6.90 (CB4-2) to 4.01% (NG135A) afterward (Supplementary Table S3). Oleic and linoleic acids accounted for approximately 74 to 80% of the total fatty acids in the noug seed oil (Table 3; Figure 3). Fatty acid profiling in noug revealed the lowest (13.90%), the highest (36.05%), and the mean (22.51%) oleic acid contents after a high-temperature experiment on the tested SC-lines (Table 3; Figure 3). The proportion of the other three major fatty acids after the high-temperature experiment were C16:0 (mean = 8.48%), C18:0 (mean = 5.18%), and C18:2 (mean = 57.31%). Hence, linoleic acid was the predominant fatty acid in all analyzed samples both before (ranging from 52.03% (NG142B) to 76.47% (CB4-10)) and after (ranging from 41.83% (CB4-2) to 67.02% (NG109)) the SC-lines were grown between 21°C to 25°C temperature in the greenhouse (Supplementary Table S3). Among the four major fatty acids, oleic and linoleic acids were influenced by the temperature to a higher degree than the other two fatty acids.

**Table 3:** Mean fatty acid content before and after a high-temperature experiment in 20 noug self-compatible (SC)-lines.

Variable	Minimum	Maximum	Mean ± SE	StDev	Variance	CV
C16:0 before	3.56	8.24	7.05 **±** 0.24	1.055	1.11	14.96
C16:0 after	7.95	9.00	8.48 **±** 0.07	0.30	0.09	3.52
C18:0 before	5.29	19.04	10.03 **±** 0.76	3.39	11.51	33.84
C18:0 after	4.01	6.90	5.18 **±** 0.12	0.56	0.31	10.73
C18:1 before	3.78	17.03	8.21 **±** 0.74	3.30	10.92	40.25
C18:1 after	13.90	36.05	22.51 **±** 1.35	6.05	36.64	26.89
C18:2 before	52.04	76.47	65.42 **±** 1.54	6.90	47.61	10.55
C18:2 after	41.83	67.02	57.31 **±** 1.37	6.14	37.65	10.71

The minimum, maximum, mean (± standard error), standard deviation (StDev), variance, and coefficient of variation (CV) for the fatty acid content (C16:0, C18:0, C18:1, C18:2) of 20 noug SC-lines.

**Figure 4:**
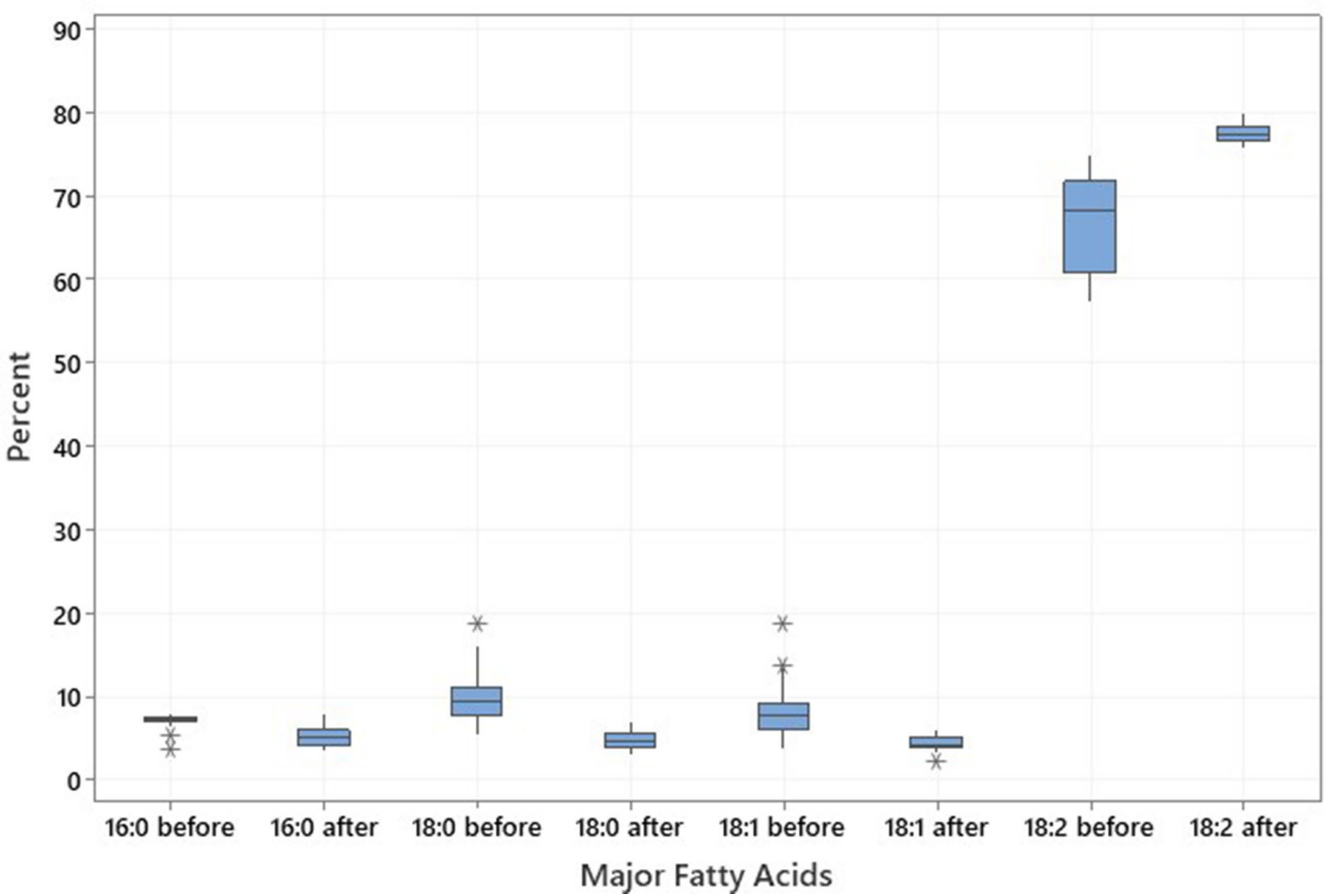
Boxplot illustrating the distribution and variation in major fatty acids palmitic acid (C16:0), stearic acid (C18:0), oleic acid (C18:1), and linoleic acid (C18:2) across the 20 noug self-compatible (SC)-lines, illustrating the changes in fatty acid profiles resulting from exposure to low temperatures of 20 noug SC lines.

The same genotypes were used for the low-temperature experiment (18-21°C) to analyze the proportion of the four major fatty acids; C16:0, C18:0, C18:1, and C18:2, of which, the two latter ones constitute approximately 75–86% of the total fatty acids in the noug seedoil (Table 4; Figure 4). The fatty acid profiling revealed the lowest (2.11%), the highest (6%), and the mean (4.43%) oleic acid contents after the low-temperature treatment on the tested SC-lines (Table 4; Figure 4). The proportion of the other three major fatty acids after the low-temperature experiment was C16:0 (5.33%), C18:0 (4.88%), and C18:2 (mean 77.5%). Linoleic acid was the predominant fatty acid in all analyzed samples both before (range: 57.41% (NG143D) to 75.02% (CB4-10)) and after (range: 75.59% (NG109) to 79.95% (NG143D)) the SC-lines were grown in low temperatures (Supplementary Table S4). Before the low-temperature experiment, oleic acid content ranged from 3.76% (CB4-10) to 18.74% (NG143D) while after treatment, it ranged from 2.11% (CB4-2) to 6% (NG135A) (Supplementary Table S4). Palmitic acid levels before the low-temperature experiment ranged from 3.68% (NG143D) to 8.01% (NG142B), and after treatment ranged from 3.53% (NG098) to 8.03% (CB4-10). Stearic acid content ranged from 5.42% (CB4-2) to 18.81% (NG142B) before treatment, decreasing to a range from 6.99% (NG109) to 3.23% (NG Bulk) afterward (Supplementary Table S4). Generally, NG142B exhibited the highest 18:0 content but the lowest 18:2 content. Conversely, CB4-10 displayed the highest 18:0 and 18:1 content but the lowest 18:2 content. Meanwhile, NG143D displayed the highest 18:1 but the lowest 16:0 content before the high-temperature experiment. Contrary, CB4-2 had the highest 18:1 content but the lowest 16:0 content, after treatment.

**Table 4:** Mean fatty acid content before and after low-temperature experiments in 20- self-compatible (SC)-lines.

Variable	Minimum	Maximum	Mean ± SE	StDev	CV
C16:0 before	3.68	8.01	7.07 ± 0.22	0.994	14.05
C16:0 after	3.53	8.03	5.33 ± 0.28	1.257	23.61
C18:0 before	5.42	18.81	9.96 ± 0.75	3.348	33.63
C18:0 after	3.23	6.99	4.88 ± 0.22	0.987	20.24
C18:1 before	3.76	18.74	8.35 ± 0.79	3.556	42.57
C18:1 after	2.11	6.00	4.43 ± 0.21	0.948	21.40
C18:2 before	57.41	75.02	67.00 ± 1.35	6.02	8.98
C18:2 after	75.59	79.95	77.50 ± 0.27	1.218	1.57

SE = Standard error; StDev = Standard deviation; CV = Coefficient of variation. Minimum, Maximum, Mean ± Standard Error (SE), Standard Deviation (StDev), and Coefficient of Variation (CV) for palmitic acid (C16:0), stearic acid (C18:0), oleic acid (C18:1), and linoleic acid (C18:2).

In the low-temperature experiment, the contents of C18:1 and C18:2 showed contrasting trends before the low-temperature experiment. Specifically, NG143D had the highest C18:1 content but with the lowest C18:2 content, while CB-10 had the lowest C18:1 but with the highest C18:2 content. Conversely, after the low-temperature experiment, NG143D had the highest C18:2 but with the lowest C18:1 content (Supplementary Table S4). Among the four major fatty acids, oleic acid was more influenced by the low temperature compared to the other three fatty acids (Supplementary Table S4).

### 3.3 Correlations between fatty acids before and after low- and high-temperature experiments

**Figure 5:**
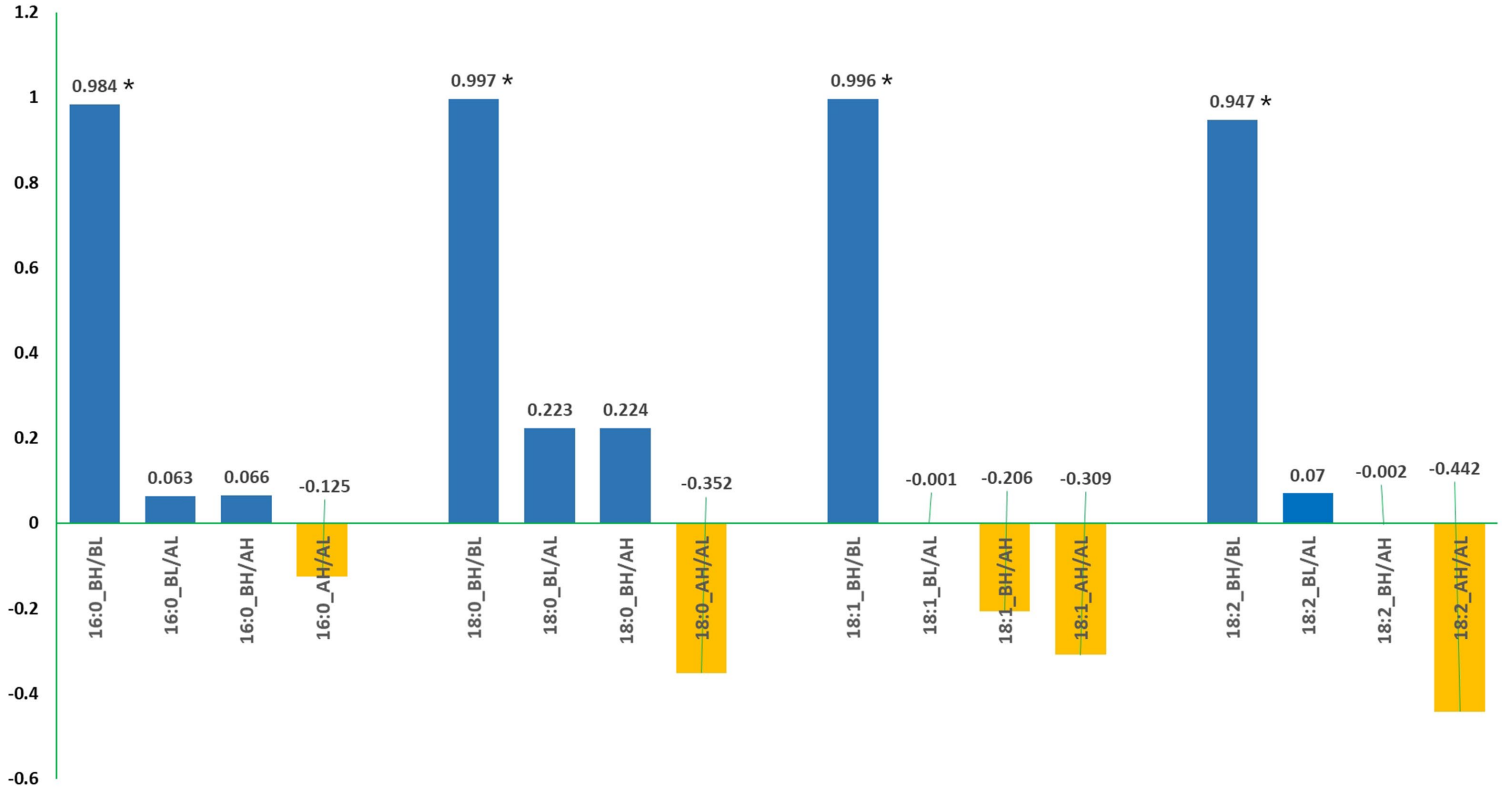
The correlation coefficient between the percentages of each fatty acid in noug genotypes before and after exposure to low-temperature (AL) and high-temperature (AH). Asterisks (*) denote significant correlations. BH indicates measurements taken before the high-temperature experiment, and BL indicates measurements taken before the low-temperature experiment.

The correlations analyzed based on data from the 20 SC-lines between all pairs of fatty acids are provided in Table 5. Linoleic acid (C18:2) had a highly significant negative correlation with C18:1 both before and after the low-temperature experiments, as well as a moderately significant negative correlation with C16:0 before the low-temperature experiment (Table 5, A). After the high-temperature experiments, linoleic acid (C18:2) maintained a highly significant negative correlation with C18:1 and a significant negative correlation with C18:0 (Table 5, B). Interestingly, 18:1 showed a significant positive correlation with 18:0 after the high-temperature experiment (Table 5). Moreover, linoleic acid (C18:2) had a moderately significant negative correlation with 18:1 before the high-temperature experiment and a highly significant negative correlation after the high-temperature experiment. Moreover, C18:1 showed a significant negative correlation with C16:0 and a significant positive correlation with C18:0 before the high-temperature experiment (Table 5, B). A paired *t*-test for four major fatty acids in noug seed oil before and after the low- and high-temperature experiments is provided in Table 6. The means of the high-temperature experiments were compared for the 20 individual plants and all plants tested (totaling 75). The analysis revealed a statistically highly significant difference (*p <* 0.001) in the means of all four major acids tested from the same individual plant, before and after the high and low-temperature experiments (Table 6). The percentages of each fatty acid in noug genotypes before and after the high-temperature experiment were not significantly correlated. Similarly, no significant correlation was found between the proportions of each fatty acid before and after the low-temperature experiment (Figure 5).

**Table 5:** Correlations among the four major fatty acids in the seed oil of noug genotypes grown under field conditions before and after exposure to (A) low-temperature and (B) high-temperature greenhouse conditions.

A	C16:0 before	C18:0 before	C18:1 before	C18:2 before
C18:0 before	0.308			
C18:1 before	−0.610**	−0.035		
C18:2 before	0.080	−0.393	−0.683***	
	C16:0 after	C18:0 after	C18:1 after	C18:2 after
C18:0 after	−0.371			
C18:1 after	−0.037	0.283		
C18:2 after	−0.095	−0.185	−0.696***	

B	C16:0 before	C18:0 before	C18:1 before	C18:2 before
C18:0 before	0.305			
C18:1 before	−0.487*	0.026		
C18:2 before	−0.042	−0.454*	−0.564**	
	C16:0 after	C18:0 after	C18:1 after	C18:2 after
C18:0 after	0.084			
C18:1 after	−0.051	0.449*		
C18:2 after	0.038	−0.545*	−0.986***	

*, **, and *** = significant at 0.05, 0.01, and 0.001 significance levels, respectively.

**Table 6:** Paired *t*-test comparing the four major fatty acids in noug seed oil before and after exposure to low- and high-temperature (T^O^) conditions, including sample sizes (N), mean values, standard deviations (StDev), standard errors of the mean (SE Mean), *t*-values, and *p*-values for each fatty acid.

Sample	*N*	Mean	StDev	SE Mean	*t*-value	*P*-value
C16:0_Before low T^O^	20	7.073	0.995	0.223	5.03	0.000
C16:0_After low T^O^	20	5.326	1.257	0.281		
C16:0_Before high T^O^	20	7.050	1.055	0.236	−5.86	0.000
C16:0_After high T^O^	20	8.485	0.300	0.067		
C16:0_Before high T^O^	75	6.85	1.05	0.236	−12.01	0.000
C16:0_After high T^O^	75	8.74	1.35	0.067		
C18:0_Before low T^O^	20	9.957	3.347	0.748	6.95	0.000
C18:0_AfterL low T^O^	20	4.875	0.985	0.220		
C18:0_Before high T^O^	20	10.029	3.393	0.759	6.09	0.000
C18:0_After high T^O^	20	5.179	0.557	0.125		
C18:0_Before high T^O^	75	8.63	2.99	0.759	−19.79	0.000
C18:0_After high T^O^	75	10.53	2.99	0.125		
C18:1_Before low T^O^	20	8.354	3.556	0.795	4.76	0.000
C18:1_AfterL low T^O^	20	4.431	0.948	0.212		
C18:1_Before high T^O^	20	8.21	3.30	0.74	−8.56	0.000
C18:1_After high T^O^	20	22.51	6.05	1.35		
C18:1_Before high T^O^	75	7.01	2.55	0.74	−43.99	0.000
C18:1_After high T^O^	75	20.22	3.06	1.35		
C18:2_BeforeL low T^O^	20	67.00	6.02	1.35	−7.76	0.000
C18:2_After low T^O^	20	77.50	1.22	0.27		
C18:2_Before high T^O^	20	65.42	6.90	1.54	3.92	0.000
C18:2_After high T^O^	20	57.31	6.13	1.37		
C18:2_Before high T^O^	75	68.29	5.90	1.54	14.35	0.000
C18:2_After high T^O^	75	58.98	4.53	1.37		

Significant differences are indicated by *p*-values less than 0.001.

## 4 Discussion

Ethiopia lacks local noug cultivars with desirable agronomic traits such as high seed yield, resistance to lodging, and shattering. Alemaw and Teklewold (21), pointed out the challenge of improving noug yield due to low variation in target traits. However, research has shown that there are noug genotypes with high seed yield, particularly large capitulum genotypes with more seeds per capitulum (16). Research has also shown that that variation in traits, such as days to flowering, days to maturity, plant height, the number of capitula per plant, the number of branches per plant, and thousand-seed weight contribute to variation in seed yield in noug. Of these, the number of branches per plant, the number of capitula per plant, and the number of seeds per capitulum are key raits that determine seed yield in noug (2, 4, 8, 15, 21). Noug also exhibits significant variation in oil content and quality (1–3). Genetic variation between and within noug populations originating from different noug growing areas is a major contributing factor to the variation in these traits along with environmental factors.

### 4.1 Effect of temperature on major fatty acid composition

Temperature is a major environmental factor that regulates the fatty acid desaturase 2 (FAD2) enzymatic activity (31). FAD2 catalyzes the synthesis of C18:2 from C18:1 (32, 33), and hence its activity subsequently affects the C18:2/C18:1 ratio (34) in seedoil. Elevated temperatures especially during the night led to a significant decline in linoleic acid levels, likely due to their influence on the activity of fatty acid desaturase enzymes especially FAD2, which is essential for the conversion of oleic acid to linoleic acid. These findings align with previous reports showing that reduced yields and changes in the fatty acid profile of sunflower oil from crops maturing in high-temperature conditions are primarily driven by heat stress impacting fatty acid biosynthesis (35, 36). Various researches have been conducted on the effect of temperature on the fatty acid composition of many oilseed crops such as sunflower (25), flax (26), soybean (27), oilseed rape (28) and safflower (29).

The composition of major fatty acids was reported to affect the nutritional value, flavor, and stability of seed oils (37). For example, the abundance of polyunsaturated fatty acids (PUFA), particularly linoleic acid, enhances the frying flavor intensity of foods, while increased oleic acid content increases oil oxidative stability (38). However, long-term storage or repeated frying use of oils with high levels of PUFA can result in off-flavors and odors because of oxidative rancidity (20). Noug seed oil has a medium to high n-6/n-3 fatty acid ratio (1–3); with linoleic acid being the most abundant PUFA (19, 20). Linoleic acid is an essential fatty acid, making it highly valuable for human consumption and for preventing nutrition-related diseases (1–3, 39). PUFAs with excess n-6 and a high n-6/n-3 ratio can promote the pathogenesis of many diseases, such as cardiovascular disease, cancer, and inflammatory diseases, whereas a low n-6/n-3 ratio has suppressive effects (39). Hence, understanding the composition of major fatty acids in oilseed crops, including noug, is vital.

The fatty acid composition of the noug SC-lines evaluated in this study corresponded well with previous studies, revealing linoleic, oleic, palmitic, and stearic acids as the major fatty acids in noug seed oils (1, 3, 7, 19). In previous studies, the levels of linoleic acid were reported to be in the range of 54 to 73% (19), 32 to 58% (40), and 72 to 78% (2, 3, 7). The oleic acid levels were reported to be in the range of 6 to 11% (7), 5.4 to 27% (19), 3.3 to 31% (1), 23 to 53% (40) and 5.2 to 9.2% (3).

Various studies showed that environmental factors, particularly temperature during seed development and maturation likely influence oilseed crops’ oil content and fatty acid composition. In this study, the highest oleic acid content was obtained from SC lines grown under 25°C/21°C day/night temperatures (high-temperature treatment). In contrast, the oleic acid content of SC lines decreased when grown under 21°C/18°C day/night temperatures (low-temperature treatment). This is consistent with the findings of Qadir et al. Who found that temperature variation affected oil and fatty acid accumulation in sunflower (41). The fatty acid profiles of sunflower, as well as other oilseed crops, are notably affected by temperature, which primarily governs the balance between oleic and linoleic acids (42, 43).

Our findings indicate that oleic acid levels were higher in SC lines planted during the spring compared to those planted in the autumn. This observation supports (43) conclusion that oleic acid content tends to rise with increasing maturation temperatures. Flagella et al. (44) also reported that fluctuations in oleic and linoleic acid levels across different sunflower hybrids might be attributed to temperature variation during the growing season. Hence, variations in oleic and linoleic acid concentrations linked to planting dates of noug may result from differing environmental conditions experienced during the growing season. Differences among sunflower hybrids were also noted regarding the minimum and maximum levels of oleic acid and the overall range of variation. As temperatures increased, the combined levels of oleic and linoleic acids also rose, while the concentration of saturated fatty acids decreased (45). This confirms the findings of (44), who found that sunflowers maturing under various ecological conditions accumulate varying levels of oleic acid.

In the present study, the levels of linoleic acid ranged from 52 to 76% in the seeds of SC lines grown under field conditions (before high-temperature experiment) but decreased to 42 to 67% (Table 3) when the same lines were grown under 25°C/21°C day/night temperatures under greenhouse conditions (after high-temperature experiment). On the other hand, oleic acid levels ranged from 4 to 17% before high-temperature experiment and increased from 14 to 36% after high-temperature experiment. The levels of palmitic and stearic acids ranged from 4 to 8% and 5 to 19% before and after high-temperature experiment, respectively. The results indicate a positive correlation between oleic acid content and temperature, and a negative correlation between linoleic acid content and temperature during growing seasons. Palmitic acid was positively correlated with stearic acid and linoleic acid after the high-temperature experiment, whereas it was negatively correlated with stearic, oleic, and linoleic acids after the low-temperature experiment. Interestingly, there was no significant correlations between palmitic and stearic acids both before and after high and low-temperature experiments, even though these fatty acids are synthesized sequentially in the fatty acid biosynthetic pathway. Moreover, palmitic acid was negatively correlated with oleic acid before both high and low-temperature experiments but was not correlated with oleic acid after the experiments.

The results of the low-temperature experiment agrees with previous reports on safflower by (29) and on noug by (1), who found a negative correlation between palmitic acid and oleic acid. However, the negative correlation of palmitic acid to steric, oleic, and linoleic acids after the low-temperature experiment might be due to the effects of low temperature on fatty acid metabolism, where certain genes contribute to high linoleic acid content at the expense of desaturation to oleic acid. The biosynthetic pathway that converts oleic acid to linoleic is responsible for this result. Linoleic and linolenic acids are generated by consecutive desaturation of oleic acid (46–48). Hence, negative correlation between oleic acid and linoleic acid is expected. The linoleic acid content of the SC lines was higher after the high-temperature experiment than after the low-temperature experiment. This is consistent with the report of (9) who indicated that the maturation of noug seeds under cooler environments contributes to an increase in linoleic acid content in noug seed oil. This shows the effect of temperature on the composition of major fatty acids in seed oil, with oleic acid being more influenced than other fatty acids.

Genotype and environment, as well as their interaction, have been previously reported to influence the composition of fatty acids in sunflower (49–51) and in noug (1, 3). Research has shown that the growing environment significantly affects noug oil fatty acid composition, with genotypes grown in low-altitude areas of Ethiopia producing elevated levels of oleic acid compared to those grown in high-altitude areas (1). Moreover (2), identified nine noug genotypes from different populations collected from three noug growing regions of Ethiopia that had elevated levels of oleic acid in their seed oil. Further breeding of these genotypes under 25°C/18°C day/night temperature conditions in a greenhouse increased the oleic acid content toup to 86% at 25°C day and 18°C night temperatures after three generations of breeding. However, such high levels of oleic acid may not be maintained in the main noug-producing areas in Ethiopia, where the temperature range is between 15°C and 23°C during the main growing season (8). Furthermore, research has shown a decrease in unsaturation when oilseed crops are grown in warmer areas due to negative correlations between the levels of linoleic and oleic acid.

Oleic and linoleic acid biosynthesis involves a series of desaturation steps leading from C18:0 to C18:1 and from C18:1 to C18:2. Hence, the increased accumulation of oleic acid in noug seed oil when grown in warmer environments could be due to either increased activity of the delta-9-desaturase enzyme, which catalyzes the production of oleic acid from stearic acid or reduced activities of the delta-12-desaturase enzyme (FAD2) that utilizes oleic acid in the production of linoleic acid (52). An increase in the oleic acid content inevitably leads to a proportional decrease in the levels of PUFAs, particularly linoleic acid in oilseed crops, as also shown in *Brassica napus* (53) and cottonseed (54, 55). The fact that Petros et al. (2) were able to obtain up to 86% oleic acid in noug seed oil and elevated levels of this fatty acid were obtained in the present study suggests the possibility of developing high oleic acid noug cultivars that could have up to 80% oleic acid in their seed oil under conducive environments (2). Furthermore, these findings suggest that temperature fluctuations can significantly influence the fatty acid composition in noug, highlighting the importance of developing temperature-resilient cultivars through breeding. Future research should focus on understanding the mechanisms through which temperature affects the fatty acid composition in noug and other oil crops in order to enhance oil quality and yield stability under varying climatic conditions, ultimately supporting the sustainability of noug production.

### 4.2 Variation in seed setting

In the present study, an average seed set of 57 seeds per capitulum was obtained, which is high compared to the previously reported about 40 seeds per capitulum (4, 8, 9) for Ethiopian noug germplasm. Seed yield is a complex, multigenic, quantitative trait influenced by environmental and epigenetic effects. Like in other crops, noug oilseed yield relies on successful flowering, seed setting, and the number of seeds reaching maturity. However, noug’s seed yield is notably low (1000–1,500 kg/ha) compared to other oilseed crops such as sesame (2000–3,500 kg/ha), irrespective of the management practices used (21). Its low productivity is associated with factors, such as self-incompatibility (9, 16), genotype-by-environment interaction (G × E), earliness (4), insect pests and diseases (4, 8, 9, 15, 21, 56).

Self-incompatibility is a major challenge in noug breeding practices because it limits the ability of plants to fertilize themselves, necessitating cross-pollination with genetically diverse individuals. This can complicate the selection and propagation of desirable traits, as breeders must ensure compatible mating systems and manage compatible pollen availability in the field, often leading to reduced seed set and inconsistent yields (16). Earliness is another key trait in noug breeding. In general, noug yields were higher in late maturing types than in early maturing types due to a tradeoff between earliness and seed yield. This is true even in most SC lines used in the present study. However, some early maturing types produced higher seeds than average mainly because of their larger capitula and more seeds per capitulum than both SI populations and other SC lines. For example, among the SC lines, CB1-14, CB2-3SFSP, and CB8-7 were early maturing but had high seed yield while CB3-9 and CB4-8 were medium maturing but had low seed-yield (Supplementary Table S2). Days to flowering, along with capitulum size and number of seeds per capitulum, are therefore very important in noug breeding programs.This is mainly because earliness is a key trait in the tropics where seasonal rainfall is short so that the crop reaches maturity while adequate precipitation is available. The fact that some SC-lines combine earliness, large capitulum size, and an increased number of seeds per capitulum is highly promising. These SC-lines can be further bred to develop cultivars with high seed and oil yields suitable for drought-prone areas with short rainy seasons.

## 5 Conclusion

The mean oleic acid content of 22.5% and linoleic acid content of 67% obtained from recently developed self-compatible lines are promising to develop high oil/high oleic and high oil/high linoleic noug cultivars through selection breeding. These high oil/high-oleic SC-lines provide several options for breeders to develop noug cultivars with optimal fatty acid composition for healthy human consumption, such as cooking oil and in fortified foods, by maintaining a balance between the oleic and linoleic contents of the oil without affecting its stability and frying flavor intensity. The high linoleic SC-lines can be used for consumption by roasting and processing the seeds in various ways. Developing molecular markers for temperature resilience in this crop is crucial for improving its nutritional profile and adaptability to climate change. Such markers enable breeders to develop resilient cultivars efficiently with sustainable yield and nutritional quality. Although most SC lines used in the present study were affected by inbreeding depression, a few were vigorous and provided high seed yields. Hence, it should be possible to develop high-yielding, self-compatible cultivars that avoid inbreeding depression by eliminating the trade-off between selfing and inbreeding depression. Such cultivars could produce higher seed yields than SI populations, increasing the crop’s contribution to food security and nutrition, and meeting the growing demand for vegetable oils. In general, noug breeding programs should focus on the development of high-yielding, self-compatible noug cultivars that have a high oleic acid content in seed oils and are well suited to warmer agro-ecologies (altitudes below 2000 meters above sea level) along with cultivars that yield high linoleic acid at higher altitudes.

## Data Availability

The original contributions presented in the study are included in the article/Supplementary material, further inquiries can be directed to the corresponding author/s.

## Footnotes

^1^ www.pfeuffer.com

^2^Minitab Inc. https://www.minitab.com/en-us/
