# Supplementary material for: Temperature affects major fatty acid biosynthesis in noug (Guizotia abyssinica) self-compatible lines
Source: Front Nutr. 2024 Dec 13;11:1511098. doi: 10.3389/fnut.2024.1511098 (PMC11673493; doi:10.3389/fnut.2024.1511098)
Supplement: Supplementary file 1 [file Table_1.DOCX]

**Supplementary Table S1.** Description of the five quantitative and seven qualitative/categorical traits of noug included in the present study.

| **Trait** | **Trait code** | **Description** |
| --- | --- | --- |
| **Quantitative** | |  |
| Days to flowering | DTF | Number of days from planting to the flowering of an individual plants |
|  |  |  |
| Plant height | PH | Height of a plant measured in cm at full flowering stage |
| Number of seeds per capitulum | NSPC | An average number of seeds per capitulum, calculated based on five capitula from each plant |
| Thousand seed weight | TSW | The weight of 100 seeds measured in grams multiplied by ten |
| **Qualitative/Categorical** |  |  |
| Flower size | FS | Three categories were given based on the diameter of fully developed flowers: 1 = small (< 3 cm); 2 = medium (3 to 4 cm); 3 = large (> 4 cm) |
| Capitulum size | CS | Three categories were given based on the cross-sectional circumference of the mature capitulum: 1 = small (< 3 cm ); 2 = medium (3 to 4 cm); 3 = large (> 4 cm ) |
| Seed color | SC | Three categories were given based on the intensity of black color of mature seeds: 1= black; 2 = brown; 3 = grey |
| Seed size | SS | Three categories were given based on the size of the mature seeds: 1 = small; 2 = medium; 3 = large |
| Seed shape | SSH | Two categories were given based on the shape of the seeds: 1 = elongated; 2 = ovoid elongated |
| Ray florets color | RFC | Two categories were given based on the color of ray florets: 1 = pale yellow; 2 = yellow |
| Pollen color | PC | Two categories were given based on the color of the pollen: 1 = pale yellow; 2 = yellow |
