# Supplementary material for: Temperature affects major fatty acid biosynthesis in noug (Guizotia abyssinica) self-compatible lines
Source: Front Nutr. 2024 Dec 13;11:1511098. doi: 10.3389/fnut.2024.1511098 (PMC11673493; doi:10.3389/fnut.2024.1511098)
Supplement: Supplementary file 2 [file Table_2.DOCX]

**Supplementary Table S2.** Descriptive statistics of days to flowering (DTF), plant height (PH), number of seeds per capitulum (NSPC), and thousand seed weight (TSW) for 14 genotype groups, each comprising three or more closely related self-compatible (SC) genotypes and for all 94 SC genotypes.

| Variable | Group | N | Mean±SE | StDev | Range | Variable | Group | N | Mean±SE | StDev | Range |
| --- | --- | --- | --- | --- | --- | --- | --- | --- | --- | --- | --- |
| DTF | Gr_01 | 3 | 87.7±0.7 | 1.2 | 87–89 | NSPC | Gr_01 | 3 | 46.7±6.7 | 11.6 | 40–60 |
|  | Gr_06 | 9 | 85.6±1.3 | 3.8 | 77–90 |  | Gr_06 | 9 | 19.7±6.3 | 18.9 | 4–65 |
|  | Gr_07 | 7 | 83.3±1.6 | 4.2 | 78–89 |  | Gr_07 | 7 | 59.1±7.3 | 19.3 | 32–79 |
|  | Gr_09 | 3 | 85.7±1.2 | 2.1 | 84–88 |  | Gr_09 | 3 | 65.3±19.1 | 33.0 | 38–102 |
|  | Gr_12 | 3 | 77.7±0.9 | 1.5 | 76–79 |  | Gr_12 | 3 | 16.0±11.1 | 19.3 | 2–38 |
|  | Gr_13 | 4 | 83.3±2.0 | 4.0 | 80–88 |  | Gr_13 | 4 | 38.5±4.5 | 9.0 | 28–50 |
|  | Gr_14 | 8 | 85.9±1.0 | 2.8 | 81–89 |  | Gr_14 | 8 | 49.1±12.3 | 34.9 | 11–114 |
|  | Gr_16 | 3 | 84.3±0.7 | 1.2 | 83–85 |  | Gr_16 | 3 | 47.7±16.8 | 29.1 | 17–75 |
|  | Gr_17 | 7 | 82.7±1.5 | 3.9 | 79–89 |  | Gr_17 | 7 | 17.3±6.7 | 17.7 | 1–50 |
|  | Gr_20 | 6 | 84.0±2.1 | 5.2 | 77–90 |  | Gr_20 | 6 | 26.2±8.7 | 21.2 | 5–58 |
|  | Gr_21 | 6 | 83.5±2.0 | 4.9 | 78–89 |  | Gr_21 | 6 | 24.0±9.1 | 22.2 | 8–63 |
|  | Gr_23 | 5 | 86.0±1.5 | 3.3 | 83–90 |  | Gr_23 | 5 | 14.8±5.9 | 13.1 | 4–37 |
|  | Gr_24 | 4 | 81.5±1.8 | 3.5 | 78–85 |  | Gr_24 | 4 | 31.8±8.7 | 17.4 | 16–56 |
|  | Gr_27 | 8 | 87.1±0.8 | 2.4 | 83–89 |  | Gr_27 | 8 | 61.8±12.4 | 35.2 | 17–110 |
|  | All | 94 | 84.0±0.4 | 4.1 | 76–90 |  | All | 94 | 34.3±2.9 | 28.1 | 1–114 |
| PH | Gr_01 | 3 | 99.3±24.9 | 43.2 | 52–136 | TSW | Gr_01 | 3 | 3.4±0 | 0.1 | 3.31–3.43 |
|  | Gr_06 | 9 | 100.6±9.5 | 28.5 | 71–150 |  | Gr_06 | 9 | 2.5±0.3 | 0.8 | 1.41–3.98 |
|  | Gr_07 | 7 | 139.7±7.7 | 20.3 | 110–176 |  | Gr_07 | 7 | 3.4±0.3 | 0.7 | 2.03–4 |
|  | Gr_09 | 3 | 135.7±2.7 | 4.6 | 133–141 |  | Gr_09 | 3 | 4.0±0.3 | 0.5 | 3.62–4.64 |
|  | Gr_12 | 3 | 115.0±12.5 | 21.7 | 92–135 |  | Gr_12 | 3 | 2.5±0.8 | 1.4 | 1.31–4.05 |
|  | Gr_13 | 4 | 105±18.3 | 36.6 | 51–129 |  | Gr_13 | 4 | 3.5±0.3 | 0.7 | 2.66–4.28 |
|  | Gr_14 | 8 | 128.7±4.8 | 13.5 | 111–145 |  | Gr_14 | 8 | 3.4±0.3 | 0.8 | 2.43–4.32 |
|  | Gr_16 | 3 | 135.8±15.3 | 26.6 | 120–167 |  | Gr_16 | 3 | 3.5±0.5 | 0.9 | 2.4–4.14 |
|  | Gr_17 | 7 | 131.6±9.0 | 23.9 | 106–169 |  | Gr_17 | 7 | 2.5±0.3 | 0.9 | 0.93–3.96 |
|  | Gr_20 | 6 | 146.9±10.3 | 25.2 | 109–182 |  | Gr_20 | 6 | 2.9±0.3 | 0.8 | 2–3.79 |
|  | Gr_21 | 6 | 130.3±8.7 | 21.3 | 109–168 |  | Gr_21 | 6 | 2.8±0.3 | 0.7 | 2.13–3.75 |
|  | Gr_23 | 5 | 140.2±11.3 | 25.3 | 113–171 |  | Gr_23 | 5 | 2.5±0.3 | 0.7 | 1.95–3.63 |
|  | Gr_24 | 4 | 120.5±25.4 | 50.8 | 47–164 |  | Gr_24 | 4 | 3.4±0.6 | 1.2 | 2.25–4.98 |
|  | Gr_27 | 8 | 124.3±6.5 | 18.5 | 92–151 |  | Gr_27 | 8 | 3.7±0.2 | 0.6 | 2.89–4.53 |
|  | All | 94 | 127.2±2.9 | 27.6 | 47–182 |  | All | 94 | 3.0±0.1 | 0.9 | 0.93–4.98 |

SE = Standard error; StDev = Standard deviation
