# Supplementary material for: Temperature affects major fatty acid biosynthesis in noug (Guizotia abyssinica) self-compatible lines
Source: Front Nutr. 2024 Dec 13;11:1511098. doi: 10.3389/fnut.2024.1511098 (PMC11673493; doi:10.3389/fnut.2024.1511098)
Supplement: Supplementary file 3 [file Table_3.DOCX]

**Supplementary Table S3.** Percent fatty acids in the seed oil of 20 SC-lines before and after the high-temperature experiment.

| **SC-lines** | **C16:0 before** | **C16:0 after** | **C18:0 before** | **C18:0 after** | **C18:1 before** | **C18:1 after** | **C18:2 before** | **C18:2 after** |
| --- | --- | --- | --- | --- | --- | --- | --- | --- |
| CB1-2 | 6.13 | 8.90 | 8.24 | 5.74 | 4.60 | 17.47 | 70.84 | 60.18 |
| CB1-17 | 6.30 | 8.38 | 8.91 | 5.24 | 13.15 | 14.47 | 63.64 | 64.96 |
| CB4-2 | 7.17 | 8.24 | 5.77 | 6.90 | 5.10 | 36.05 | 70.52 | 41.83 |
| CB4-6 | 7.18 | 8.63 | 7.27 | 5.36 | 4.80 | 25.40 | 71.15 | 53.72 |
| CB4-9 | 7.84 | 8.27 | 9.81 | 4.81 | 13.85 | 19.72 | 60.07 | 59.22 |
| CB4-10 | 7.31 | 8.21 | 5.29 | 4.85 | 3.78 | 32.00 | 76.47 | 50.14 |
| CB4-13 | 7.15 | 7.95 | 6.29 | 5.16 | 6.00 | 17.93 | 68.15 | 61.83 |
| NG089 | 7.43 | 8.66 | 10.20 | 5.07 | 8.33 | 19.03 | 68.89 | 60.63 |
| NG095 | 7.19 | 8.35 | 9.21 | 5.46 | 8.45 | 24.00 | 69.76 | 56.69 |
| NG098 | 7.67 | 8.82 | 9.97 | 5.65 | 9.15 | 19.18 | 64.33 | 60.62 |
| NG109 | 7.60 | 8.68 | 16.46 | 4.96 | 6.83 | 13.90 | 63.04 | 67.02 |
| NG120 | 7.68 | 8.21 | 14.31 | 5.45 | 6.55 | 24.50 | 66.37 | 55.20 |
| NG123 | 7.34 | 9.00 | 11.04 | 4.96 | 7.68 | 24.57 | 72.36 | 55.14 |
| NG124 | 5.30 | 8.58 | 10.20 | 4.87 | 8.20 | 19.01 | 75.51 | 61.35 |
| NG135A | 8.24 | 8.26 | 11.05 | 4.01 | 6.20 | 17.11 | 66.43 | 63.81 |
| NG139D | 7.59 | 8.04 | 8.52 | 4.65 | 9.13 | 18.91 | 55.83 | 60.36 |
| NG140 Bulk | 7.18 | 8.79 | 11.35 | 5.10 | 7.90 | 22.15 | 60.60 | 58.06 |
| NG142B | 7.84 | 8.75 | 19.04 | 5.23 | 7.48 | 32.96 | 52.04 | 47.04 |
| NG143C | 7.30 | 8.68 | 9.68 | 4.86 | 10.00 | 25.69 | 55.63 | 55.07 |
| NG143D | 3.56 | 8.29 | 7.97 | 5.24 | 17.03 | 26.23 | 56.74 | 53.36 |
