# Supplementary material for: Temperature affects major fatty acid biosynthesis in noug (Guizotia abyssinica) self-compatible lines
Source: Front Nutr. 2024 Dec 13;11:1511098. doi: 10.3389/fnut.2024.1511098 (PMC11673493; doi:10.3389/fnut.2024.1511098)
Supplement: Supplementary file 4 [file Table_4.DOCX]

**Supplementary Table S4.** Percent fatty acids in the seed oil of 20 SC-lines before and after the low-temperature experiment.

| **SC-lines** | **C16:0 before** | **C16:0**  **after** | **C18:0 before** | **C18:0**  **after** | **C18:1 before** | **C18:1**  **after** | **C18:2 before** | **C18:2**  **after** |
| --- | --- | --- | --- | --- | --- | --- | --- | --- |
| CB1-2 | 6.43 | 6.14 | 8.40 | 4.10 | 4.80 | 4.00 | 74.51 | 78.22 |
| CB1-17 | 6.40 | 4.42 | 9.06 | 6.27 | 13.46 | 5.77 | 64.89 | 76.56 |
| CB4-2 | 7.32 | 6.27 | 5.42 | 4.04 | 5.33 | 2.11 | 72.15 | 79.84 |
| CB4-6 | 7.44 | 7.21 | 7.40 | 3.33 | 4.84 | 3.33 | 74.87 | 77.67 |
| CB4-9 | 7.79 | 4.18 | 9.91 | 5.78 | 13.71 | 4.24 | 60.94 | 78.76 |
| CB4-10 | 7.68 | 8.03 | 5.71 | 4.65 | 3.76 | 5.36 | 75.02 | 77.49 |
| CB4-13 | 7.12 | 7.60 | 6.28 | 5.17 | 6.23 | 4.64 | 70.59 | 75.61 |
| NG089 | 7.03 | 5.01 | 9.52 | 4.47 | 8.68 | 4.98 | 70.13 | 77.37 |
| NG095 | 7.05 | 4.39 | 9.06 | 5.53 | 8.60 | 3.98 | 68.26 | 78.39 |
| NG098 | 7.49 | 3.53 | 9.61 | 3.79 | 9.10 | 3.28 | 66.76 | 77.99 |
| NG109 | 7.52 | 4.05 | 16.23 | 6.99 | 6.52 | 4.47 | 66.27 | 75.59 |
| NG120 | 7.58 | 5.66 | 14.27 | 4.48 | 6.48 | 4.61 | 68.40 | 76.99 |
| NG123 | 7.44 | 5.58 | 11.42 | 5.69 | 7.41 | 4.19 | 68.70 | 77.21 |
| NG124 | 5.46 | 4.67 | 9.80 | 4.17 | 8.22 | 4.18 | 75.02 | 78.33 |
| NG135A | 7.99 | 4.01 | 11.24 | 5.25 | 6.21 | 6.00 | 68.96 | 76.73 |
| NG139D | 7.49 | 4.06 | 8.51 | 4.73 | 9.20 | 4.14 | 60.63 | 77.62 |
| NG140 Bulk | 7.20 | 5.96 | 11.30 | 3.23 | 8.09 | 5.47 | 60.81 | 76.05 |
| NG142B | 8.01 | 5.35 | 18.81 | 4.52 | 7.52 | 5.24 | 57.98 | 76.21 |
| NG143C | 7.35 | 5.42 | 9.61 | 5.85 | 10.18 | 5.11 | 57.66 | 77.37 |
| NG143D | 3.68 | 4.99 | 7.57 | 5.46 | 18.74 | 3.53 | 57.41 | 79.95 |
